# Antioxidant activity and mechanism of commercial Rama Forte persimmon fruits (*Diospyros kaki*)

**DOI:** 10.7717/peerj.5223

**Published:** 2018-07-25

**Authors:** Luana T. Dalvi, Daniel C. Moreira, Antonio Alonso, Isa G.J. de Avellar, Marcelo Hermes-Lima

**Affiliations:** 1 Departamento de Biologia Celular, Universidade de Brasília, Brasília, Distrito Federal, Brazil; 2 Departamento de Nutrição, Universidade de Brasília, Brasília, Distrito Federal, Brazil; 3 Área de Morfologia, Faculdade de Medicina, Universidade de Brasília, Brasília, Distrito Federal, Brazil; 4 Instituto de Física, Universidade Federal de Goiás, Goiânia, Goiás, Brazil; 5 Instituto de Química, Universidade de Brasília, Brasília, Distrito Federal, Brazil

**Keywords:** Fruit maturation, Oxidative stress, Polyphenol, Lipid peroxidation, Tannin

## Abstract

This study aimed to characterize the antioxidant properties of Rama Forte persimmon, a tannin-rich fruit variety produced in Brazil. Extracts prepared with lyophilized pulps from fruits obtained in local markets were analyzed individually to evaluate the extent of antioxidant protection and investigate the antioxidant mechanism. Iron-mediated hydroxylation of 5,5-dimethyl-1-pirrolidine-N-oxide, determined by electron paramagnetic resonance (EPR), and oxidative degradation of 2-deoxyribose (2-DR) were inhibited by fruit extracts in a dose-dependent manner. There was a considerable individual variability in inhibition of 2-DR degradation by individual fruits. Higher protection of 2-DR degradation (by the extracts) was observed in Fe(III)-citrate/ascorbate in comparison with Fe(III)-EDTA/ascorbate system; however, antioxidant effectiveness of fruit extracts was not diminished by increasing EDTA concentration by 10-fold. Other competition experiments using the 2-DR assay (varying pre-incubation time and 2-DR concentration) indicated that protection comes mainly from free radical scavenging, rather that metal chelation antioxidant activity. Persimmon extracts prevented iron-mediated lipid peroxidation in rat liver homogenates, which correlated significantly with the inhibition of 2-DR oxidation. Finally, sugar content of individual fruits correlated inversely with inhibition of 2-DR degradation, which could indicate that maturation decreases soluble antioxidant concentration or efficiency. In conclusion, lipid peroxidation, 2-DR and EPR experiments indicated that extracts from commercial fruits showed mainly radical-scavenger activity and relevant antioxidant activity.

## Introduction

Special interest in the benefits provided by fruits and vegetables has appeared since epidemiological studies showed some degree of correlation among their consumption and reduced risk for coronary heart disease, hypertension and stroke, cancer, diabetes and other degenerative and chronic diseases (Hertog et al., 1993; Liu et al., 2000; Boeing et al., 2012; Oyebode et al., 2014; Wang et al., 2014; Micha et al., 2017). Phytochemicals, such as polyphenolic compounds, carotenoids, ascorbate and vitamin E received much attention, mainly due to their antioxidant activity and the relevant role they would play in prevention and treatment of several human diseases (Khan, Afaq & Mukhtar, 2008; Wolfe et al., 2008; Zhang & Tsao, 2016). Antioxidants may scavenge reactive free radicals and inhibit radical chain reactions or may act as metal chelators preventing the radical formation. More recently, the role of antioxidants in cellular signaling has been increasingly considered (Forman, Davies & Ursini, 2014). The *in vitro* antioxidant activity of numerous plant extracts has been well described in the literature and most studies focused mainly on the reducing capacity of different extracts using the Folin-Ciocalteu/Folin-Denis, ABTS or DPPH assays (Wang, Cao & Prior, 1996; Velioglu et al., 1998; Katsube et al., 2004; Miliauskas, Venskutonis & Van Beek, 2004; Gu et al., 2008; Gulçin, 2012; Vinha et al., 2012; Tan & Lim, 2015). Since chelation of transition metals by polyphenols may interfere with free radical production (Lopes, Schulman & Hermes-Lima, 1999; Andrade et al., 2005; Andrade et al., 2006; Genaro-Mattos et al., 2015; Dalvi et al., 2017), metal chelating capacity of plant extracts may be of pivotal importance, especially when analyzing plant extracts rich in polyphenols—well-known metal-binding molecules (Perron & Brumaghim, 2009).

Persimmon fruits (*Diospyros kaki*) are rich in antioxidants such as ascorbic acid, carotenoids and various polyphenols, including tannins (Gorinstein et al., 2001; Suzuki et al., 2005; Butt et al., 2015; Favati et al., 2018; Maulidiani et al., 2018). There are several types of persimmon cultivars with variable astringency, a property related to the tannin content. The total phenolic content in 100 g of dry persimmon pulp can reach 85 mg in astringent fruits, but it may be 4–6 times lower in non-astringent persimmons (Suzuki et al., 2005). Persimmon fruits improve the lipid profile and reduce levels of lipid peroxidation in rats under high-fat diet (Gorinstein et al., 1998; Matsumoto et al., 2006; Fushimi et al., 2015). Studies *in vitro* demonstrated that persimmon fruits have the ability to scavenge DPPH and ABTS radicals, prevent LDL oxidation and inhibit oxidative damage to human leukocytes (Katsube et al., 2004; Yokozawa et al., 2007; Gu et al., 2008; Jang et al., 2010; Grygorieva et al., 2018). Persimmon has a greater antioxidant capacity than blackberry, blueberry and strawberry (measured by the ABTS assay), but it is less effective against iron-induced lipid peroxidation (García-Alonso et al., 2004). Many other studies demonstrated that persimmon extracts have antioxidant activities, but the detailed antioxidant mechanisms remain to be investigated (Han et al., 2002; Kondo, Yoshikawa & Katayama, 2004; Lee, Cho & Yokozawa, 2008; Vinha et al., 2012; Maulidiani et al., 2018).

Brazil is among the top five persimmon producers in the world (Food and Agriculture Organization of the United Nations, 2017). The Rama Forte cultivar is an astringent, seedless, tannin rich variety, commonly commercialized and consumed there. Before commercialization, astringent persimmon cultivars undergo a treatment to reduce their astringency, by means of CO_2_ gas or vapors of ethanol or ethylene, which diminishes soluble tannin content to make fruits more palatable (Kato, 1990; Del Bubba et al., 2009). The ethylene method has been the most commonly used in Brazil for Rama Forte cultivars (Muñoz, 2002; Vitti, 2009). The present study aims to determine the antioxidant properties of extracts from persimmon fruits from Brazilian markets, determining whether their mechanism of action is free radical scavenger, metal-chelating antioxidant or both. The influence of fruit maturation and astringency removal on the persimmon antioxidant activity is also discussed. We employed individual commercial persimmons (those available for consumption by people) to analyze whether fruit variability affects antioxidant activity.

## Materials and Methods

### Reagents, solutions and fruits

Ascorbic acid, 2-deoxyribose (2-DR), ferrous ammonium sulfate, N-[2-hydroxyethyl]-piperazine-N′-[2-ethanesulfonic acid] (HEPES), thiobarbituric acid (TBA), mono and dibasic potassium phosphate, ethylenediaminetetraacetate (EDTA), tannic acid, and 5,5-dimethyl-1-pirrolidine-N-oxide (DMPO) were purchased from Sigma (St. Louis, MO, USA). Hydrogen peroxide (H_2_O_2_, 30%) was purchased from Merck. Ferric chloride hexahydrate, sodium citrate and phosphoric acid from various sources were analytical grade. Stock solutions of ferrous ammonium sulfate and H_2_O_2_ were freshly prepared prior to each experiment. Stock solutions of 1% w/v TBA were prepared in 50 mM NaOH and used within two days.

Rama Forte persimmon fruits, from a single production origin (Guarema—São Paulo, Brazil), were bought at a fruit distribution and marketing center in Brasília (CEASA-DF, Brazil) in three distinct purchases in April of 2007. Twenty persimmons were selected to the study according to homogeneity in aspect, integrity of peel, softness of flesh and the color (red). Immediately after each purchase, the selected fruits (seven from the first purchase, eight from the second purchase and five from the third purchase) were thoroughly rinsed with Milli-Q water, weighed and peeled. Fruits were labeled individually with numbers #1–#20. After weighing, the pulps were frozen in liquid nitrogen and lyophilized. Dried material, corresponding to 52% (in average) of the frozen pulp, was triturated in a porcelain mortar. The pulp powders were kept at −20 °C until the determinations (done within 2–3 months).

Persimmon aqueous solutions were prepared by maceration of 10 mg of the pulp powder per milliliter of Milli-Q water with a glass manual tissue grinder. Resulting suspensions were centrifuged for 30 min at 13,000 rpm and the supernatants were used in the experiments. Persimmon stock solutions were prepared daily, a few minutes before use.

### The 2-deoxyribose degradation assay

This method is based on the determination of malonaldehyde, an oxidation product of 2-deoxyribose, by measurement of the colored condensation product with TBA at 532 nm (Andrade et al., 2006). In typical reactions, hydroxyl radical was generated by Fe(III)-citrate (1:1) or Fe(III)-EDTA (1:1) plus ascorbate in buffered media (20 mM phosphate, pH 7.2) (see reactions 1–4). After 10 min pre-incubating persimmon extracts with the Fe(III) complex (using citrate or EDTA as the ligand) and 2-deoxyribose, reactions were initiated by ascorbate addition, carried out for 30 min at room temperature (24–25 °C) and stopped by the addition 0.5 mL 4% (v/v) phosphoric acid followed by 0.5 mL TBA solution. Solutions were incubated for 15 min in boiling water bath and allowed to cool to room temperature before absorbance measurement. Each experimental result was corrected for its correspondent blank reaction (called “time zero” blank) to eliminate the background interference. In the “time zero” blank, ascorbate was added to the media after phosphoric acid and TBA addition (Andrade et al., 2006). This also discounts for 2-DR oxidation by Fe(III) in the analytical phase of the assay (Genaro-Mattos et al., 2009).

(1) \begin{eqnarray*}& & \text{Fe(III)-EDTA/citrate}+\text{ascorbate}\rightarrow \text{Fe(II)-EDTA/citrate}+\text{ascorbyl}\end{eqnarray*}

(2) \begin{eqnarray*}& & \text{Fe(II)-EDTA/citrate}+{\mathrm{O}}_{2}\rightarrow \text{Fe(III)-EDTA/citrate}+{\mathrm{O}}_{2}^{-\bullet }\end{eqnarray*}

(3) \begin{eqnarray*}& & 2{\mathrm{O}}_{2}^{-\bullet }+2{\mathrm{H}}^{+}\rightarrow {\mathrm{H}}_{2}{\mathrm{O}}_{2}+{\mathrm{O}}_{2}\end{eqnarray*}

(4) \begin{eqnarray*}& & {\mathrm{H}}_{2}{\mathrm{O}}_{2}+\text{Fe(II)-EDTA/citrate}\rightarrow \text{Fe(III)-EDTA/citrate}+{\mathrm{OH}}^{-}+{}^{\bullet }\mathrm{OH}\end{eqnarray*}

(5) \begin{eqnarray*}& {& }^{\bullet }\mathrm{OH}+\text{2-deoxyribose}\rightarrow \text{degradation products}.\end{eqnarray*}

### Lipid peroxidation assay

Adult Wistar rats were provided by professor Egle de Almeida’s Laboratory in University of Brasilia, Brazil. They were housed individually at 21–23 °C, 12 h light/dark cycles with water and food ad libitum. Rats were euthanized by cervical dislocation. Livers were dissected, rinsed with cold 0.9% (w/v) NaCl, flash frozen in liquid nitrogen and stored at −80 °C until used. These procedures were approved by the Ethics Committee for the Use of Animals of the Institute of Biological Sciences, University of Brasilia, Brazil (UnB-DOC 120380/2009). Rat liver homogenates were prepared in buffer solution (125 mM KCl, 100 mM HEPES, pH 7.2) in the proportion of 1:4 (w/v). Tissue was macerated in glass tissue grinder in ice bath, centrifuged (400 rpm, 15 min) at low temperature and the supernatant was used in the assays. Fe(III)-citrate/ascorbate mixture were used for oxyradical generation. Reactions were done in 200 µL (total volume) and started by the addition of Fe(III)-citrate. Reactions were interrupted by addition of 7% (v/v) phosphoric acid solution (100 µL) and 1% (w/v) TBA solution (200 µL). Reaction media were incubated in boiling water bath (98 °C) for 15 min, then allowed to cool. Absorbance of TBARS is the difference between absorbance at 532 nm and 600 nm, the later corresponding to the interference from the liver homogenate (Hermes-Lima, Willmore & Storey, 1995). Negative controls were performed with butylated hydroxytoluene (BHT). A “time zero” blank was also performed for each experimental condition to eliminate the background absorbance of our chemical reagents (see the ‘The 2-deoxyribose Degradation Assay’ for more details). Lipid peroxidation experiments were performed using persimmon extracts at 2 or 4 mg/mL.

### EPR measurements

A Bruker ESP 300 spectrometer (Bruker, Rheinstetten, Germany) equipped with an ER 4102 ST resonator was used to perform the EPR measurements. The instrument parameters were as follows: microwave power, 2 mW; modulation frequency, 100 kHz; modulation amplitude, 1.0 G; magnetic field scan, 100 G; sweep time, 168 s; and detector time constant, 41 ms; receive gain, 10^5^. All measurements were performed at room temperature (24–26 °C). Hydroxyl radicals formed by Fenton reagents (50 µM Fe(II) and 100 µM H_2_O_2_) in buffered media (10 mM phosphate, pH 7.2) were trapped by DMPO (20 mM) (Zalomaeva et al., 2007). The effect of different concentrations of persimmon extract was investigated against DMPO hydroxylation. Spectra were acquired until 3 min after reaction started and the quantification of DMPO-OH adduct was done by measurement of first line resonance peak height diminished of EPR baseline signal.

### Determination of sugar content

Sugar content was determined using the phenol–sulfuric acid assay adapted from DuBois et al. (1956). Sugars from 10 mg of dried persimmon were extracted in 2 mL of ethanol 80% after vigorous shaking and incubation for 20 min at 80 °C. Sugar extracts were diluted 25 times in water and 0.5 mL of each samples were added in 0.5 mL of 5% phenol in water followed by the addition of 2.5 mL of concentrated sulfuric acid. The amount of sugar was analyzed at 490 nm and determined using a glucose standard curve previously constructed as reference.

### Statistical analyses

Analysis of data was performed using SPSS statistical analysis software (version 19). For correlation analyses individual 2-DR assay, lipid peroxidation and sugar content values were evaluated for the assumption of normality by the D’Agostino & Pearson test, before applying parametric (Pearson correlation) or nonparametric (Spearman correlation) tests. The antioxidant effect among fruits was analyzed using ANOVA and the post-analysis was performed by the Student-Newman-Keuls (SNK) test employing *P* < 0.05 for significant differences.

## Results and Discussion

### DMPO-OH spin-trapping study

**Figure 1:**
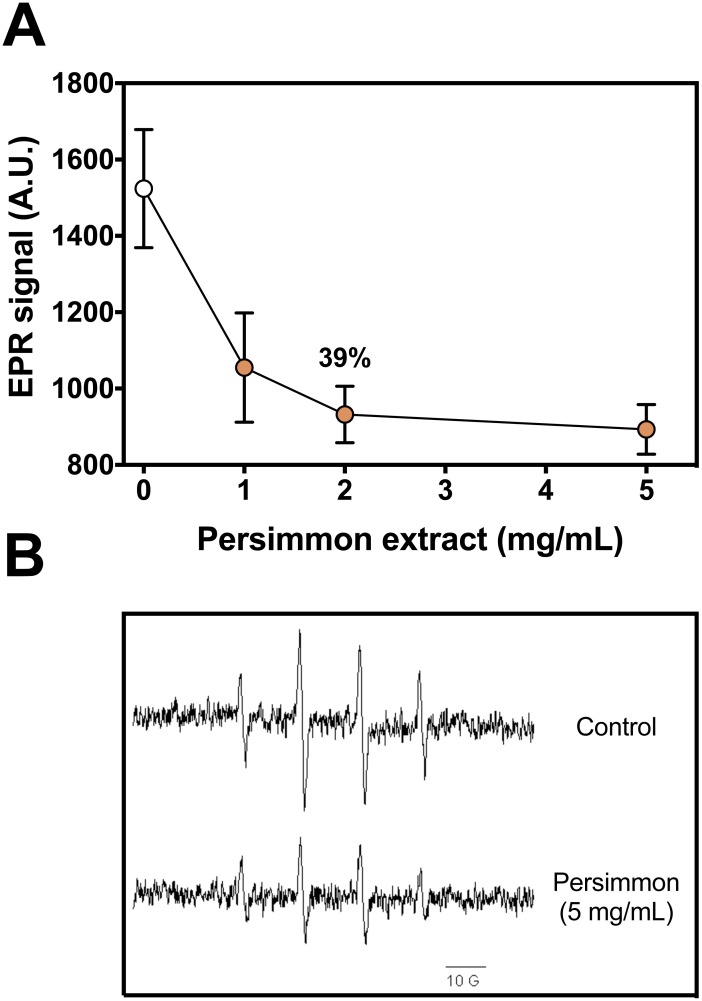
Radical scavenging activity of persimmon extract. (A) Effect of persimmon extracts (average values from experiments using fruits #4 and #10 individually) on DMPO-OH adduct formation measured by EPR method. Reaction media were buffered (10 mM phosphate, pH 7.2) and contained 50 µM Fe(II), 100 µM H_2_O_2_, 20 mM DMPO and fruit extracts (0–5 mg/mL). Reactions were initiated by Fe(II) addition to media. Values are means ± SD (*n* = 3). (B) EPR signal of DMPO-OH adduct formed in absence and presence of 5 mg/mL persimmon extract. A.U. stands for arbitrary units of signal intensity.

DMPO-OH adduct formation from Fenton reagents was reduced by up to 40% in the presence of persimmon fruit extracts (Fig. 1). Increasing concentrations of persimmon extract in the reaction media induced a gradual decrease in the intensity of the EPR adduct signal. Inhibition of the adduct formation could result from two reactions: (i) iron chelation by components in persimmon extract, reducing the production of hydroxyl radicals and (ii) free radical scavenging by persimmon extract and deactivation hydroxyl radical after its formation from Fenton reaction. The inhibition mechanism was further investigated by the 2-DR oxidative degradation assay and by lipid peroxidation experiments.

### The 2-DR degradation assay

**Figure 2:**
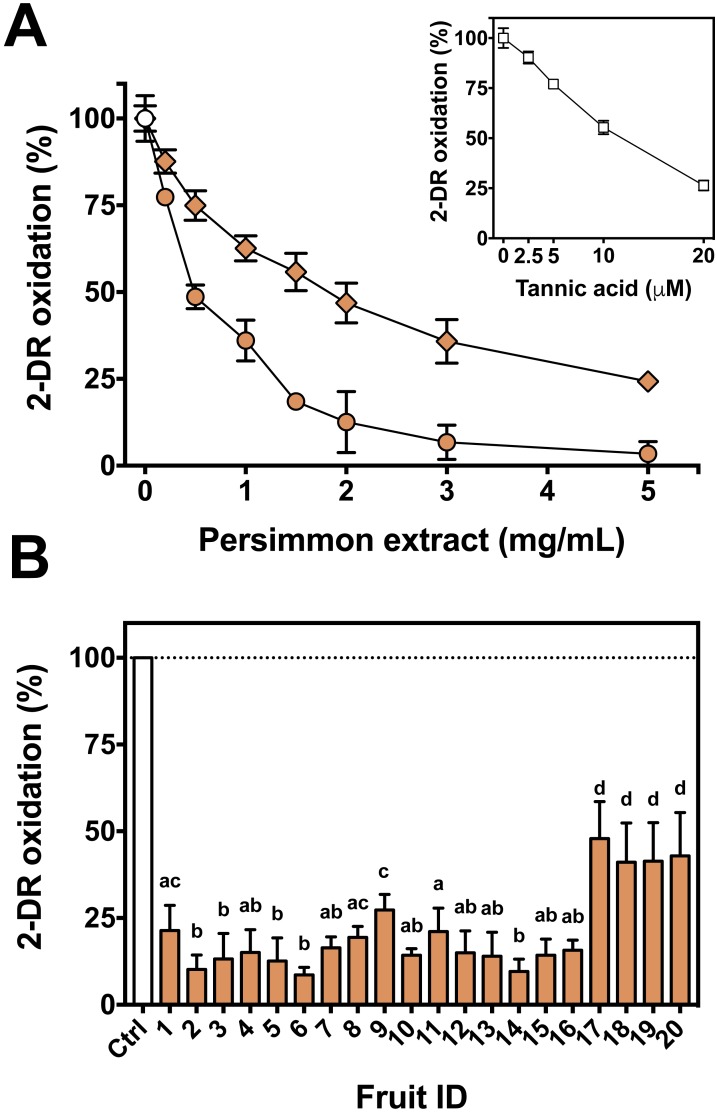
Antioxidant activity of persimmon extract against 2-DR oxidation. (A) Effect of persimmon extract concentration, using fruits #3 (circles) and #17 (diamonds) on 2-DR degradation. Reaction media also contained 20 mM phosphate buffer (pH 7.2), 5 mM 2-DR, 50 µM Fe(III)-citrate (1:1 ratio) and 0.5 mM ascorbate. Media was pre-incubated for 10 min before the addition of ascorbate and the reaction was carried out for 30 min at room temperature. Absorbance at 532 nm for control values (without extracts) was 0.150 in average. The inset shows the effect of tannic acid on 2-DR oxidation under the same experimental conditions. (B) Variability on the antioxidant effect presented by 2 mg/mL persimmon extracts on 2-DR oxidative degradation assay induced by Fe(III)-citrate and ascorbate as described above. Values are means ± SD (*n* = 6). Significant difference among results are indicated by distinct letter labels on top (*p* < 0.05; ANOVA/SNK).

First, the oxidative 2-DR degradation assay with Fe(III)-citrate and ascorbate was performed with increasing concentrations of persimmon extracts from two specific fruits, #3 and #17, chosen randomly among the 20 available (Fig. 2A). Antioxidant protection from the two extracts differed significantly, with IC_50_ values of approximately 0.8 mg/mL for fruit #3 and 1.7 mg/mL for fruit #17. Because of such divergence between fruits, the extracts of each remaining fruits were individually tested at a single concentration (2 mg/mL). Fruits #1 to #16 had similar protective effect on 2-DR oxidation, while fruits #17 to #20 had lower antioxidant activity than the others (Fig. 2B). Thus, fruits #1 to #16 were selected to the subsequent studies. In addition, the inset to Fig. 2 compares the antioxidant action of tannic acid with fruit extracts.

**Figure 3:**
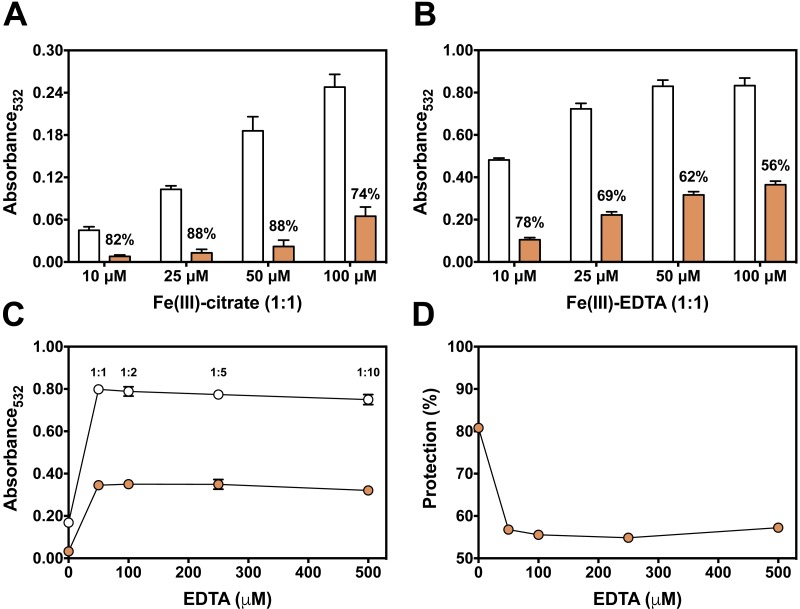
Effect of chelator affinity on antioxidant activity of persimmon extract in 2-DR oxidation assays. Antioxidant effect of persimmon extracts (average values from experiments using fruits #2, #6 and #14 individually) against either Fe(III)-citrate (A) or Fe(III)-EDTA (B) induced 2-DR oxidation. Reaction media also contained 20 mM phosphate buffer (pH 7.2), 5 mM 2-DR 0.5 mM ascorbate and the iron complex. Ascorbate was added to the media after 10 min pre-incubation of other components. Incubation time was 30 min. (C) shows the effect of EDTA concentration in 2-DR oxidative degradation in the absence (empty circles) or presence of 2 mg/mL of persimmon extracts (filled circles) (average values from experiments using fruits #7 and #12 individually) in 20 mM phosphate buffered media (pH 7.2) containing 5 mM 2-DR, 50 µM Fe(III) and EDTA (0–500 µM); ascorbate (0.5 mM) was added after 10 min preincubation Values are means ± SD (*n* = 6). Fe:EDTA ratios are indicated by the number above symbols. (D) shows the relative protection in relation to EDTA concentration.

We next used the 2-DR assay to investigate if the antioxidant mechanism of persimmon extracts arises from free radical scavenging, metal chelation or a combination of both. To do so, the assay was performed in media containing ascorbate plus various concentrations of Fe(III)-citrate or Fe(III)-EDTA as ROS generating systems. Persimmon extracts were more effective against 2-DR degradation in the presence of Fe(III)-citrate (Fig. 3A) than Fe(III)-EDTA (Fig. 3B). At 25 µM Fe(III), antioxidant protection decreased from 88% in the presence of citrate to 69% in the presence of EDTA. A similar effect on 2-DR protection was observed with 50 and 100 µM iron. In both experiments, antioxidant protection from extracts decreased with increasing iron concentrations, more prominently in the experiments with iron-EDTA.

At a first glance, the higher antioxidant effect of persimmon extracts in the iron-citrate oxidant system indicates that components of persimmon extracts would behave as typical metal chelators with antioxidant activity. Such premise is based on the difference between the formation constants of the Fe(III)-citrate and Fe(III)-EDTA complexes, which are 10^8^ and 10^25^ respectively (Roche, 1990). Therefore, a much more favorable equilibrium for the removal of iron from citrate is expected. The substitution of redox–active complexes (Fe(III)-citrate and Fe(III)-EDTA) by iron complexes less prone to mediate ROS generation (iron-polyphenol) is a plausible explanation for the antioxidant activity. In that regard, polyphenolic compounds from persimmon pulp (Gorinstein et al., 2001; Suzuki et al., 2005), would account for the supposed chelator-type antioxidant activity. We have previously demonstrated that the polyphenols tannic acid and ellagic acid also presented a highly protective effect when 2-DR degradation was induced by Fe(III)-citrate than by Fe(III)-EDTA (Andrade et al., 2006; Dalvi et al., 2017).

**Figure 4:**
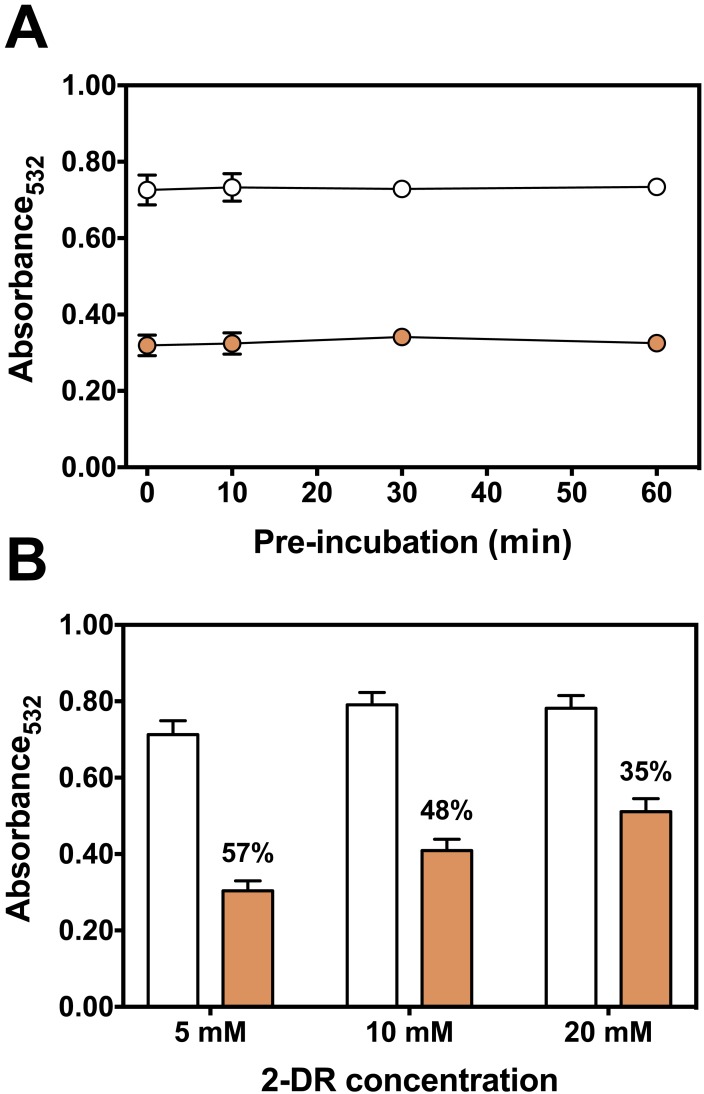
Effect of pre-incubation time and 2-DR concentration on antioxidant activity of persimmon extract in 2-DR oxidation assays. (A) Effect of pre-incubation time of persimmon extracts with 5 mM 2-DR and 50 µM Fe(III)-EDTA (1:1 ratio) in 20 mM phosphate buffered media (pH 7.2) against 2-DR degradation. Assays were performed in the absence (empty circles) or in the presence of 2 mg/mL persimmon extracts (filled circles; average values from experiments using fruits #7, #14 and #16 individually). After pre-incubation (0–60 min), reactions were initiated with 0.5 mM ascorbate addition and carried out for 30 min. (B) Effect of 2-DR concentration on the antioxidant activity of persimmon extracts. Columns represent results in the absence (empty columns) or in the presence of 2 mg/ml persimmon extracts (filled columns; average values from experiments using fruits #7, #13 and #14 individually). Reaction media also contained 20 mM phosphate buffer (pH 7.2), 0.5 mM ascorbate, 50 µM Fe(III)-EDTA (1:1 ratio) and 2-DR (5–20 mM). Media components were pre-incubated for 10 min before ascorbate addition. Results shown in (A) and (B) are means ± SD (*n* = 6–12).

To analyze a putative chelating activity, EDTA concentration was increased from 50 to 500 µM while maintaining Fe(III) concentration at 50 µM. Increasing EDTA concentrations, however, did not induce a decrease in the effect of persimmon extracts in preventing 2-DR damage (Fig. 3C). This observation does not fully support the iron-chelating hypothesis in metal-mediated ROS production. Under these conditions, the inhibitory effect of persimmon extracts on 2-DR degradation remained about 55% for any Fe(III) to EDTA ratio (Fig. 4), except for no EDTA addition, in which case iron forms a complex with 2-DR and a site-specific mechanism of oxidation takes place (Halliwell & Gutteridge, 2015). Differently from findings in persimmon extracts, polyphenols that present a chelating mechanism, such as tannic acid and ellagic acid, had their antioxidant effectiveness reduced when Fe(III)-EDTA ratio had increased from 1:1 to 1:10 (Andrade et al., 2006; Dalvi et al., 2017). The aqueous extract of *Mangifera indica* L (Vimang®) was also less effective in preventing 2-DR degradation when EDTA concentration raised from 50 to 250 µM, indicating a major contribution of chelating action to the antioxidant effect of Vimang® (Pardo-Andreu et al., 2006).

Longer pre-incubation intervals, which correspond to the period before adding ascorbate to initiate the reaction, were also tested to further investigate the metal chelating ability of persimmon extracts. Typical chelators with antioxidant activity are more effective after extended pre-incubation periods (Maurício et al., 2003; Andrade et al., 2006; Dalvi et al., 2017). Extended pre-incubation of Fe(III)-EDTA with persimmon extracts that could favor ligand exchange had no influence on the 2-DR degradation induced by the iron complex plus ascorbate (Fig. 4A). This result further indicates that persimmon extracts have a poor chelating activity.

Another way to test the antioxidant mechanism is to increase the concentration of the target molecule (i.e., 2-DR). Chelating agents inhibit the 2-DR damage by preventing ^•^OH formation. Scavenging agents, on the other hand, scavenge ^•^OH once they are formed. Increasing the amount of the target molecule (2-DR) should not affect the antioxidant protection of chelating agents but should decrease the protection of scavenging molecules (Lopes, Schulman & Hermes-Lima, 1999; Andrade et al., 2006). Figure 4B shows the effect of increasing concentrations of 2-DR in the extract’s antioxidant action. In the absence of persimmon extract, the levels of 2-DR oxidation product (malonaldehyde) do not change significantly when 2-DR concentration increased from 5 to 20 mM, indicating that at 5 mM (2-DR concentration used in standard experimental conditions) the 2-DR is not a limiting reactant. Therefore, malonaldehyde formation in our conditions is not limited by 2-DR concentration, but by ^•^OH formation. Persimmon extracts protection against oxidative damage of 2-DR induced by Fe(III)-EDTA plus ascorbate decreased from 57% at 5 mM 2-DR to 35% at 20 mM. Such decreased antioxidant protection indicates a typical hydroxyl radical scavenging activity. In summary, the results indicate that the antioxidant activity of persimmon extracts, as determined by 2-DR assays, stems from radical scavenger mechanisms and the metal-chelating property has a minor contribution (see ‘Diversity of Antioxidant Activity Among Fruits and Correlations between 2-DR Assay and Lipid Peroxidation or Sugar Content’, below).

### Iron-mediated lipid peroxidation

**Figure 5:**
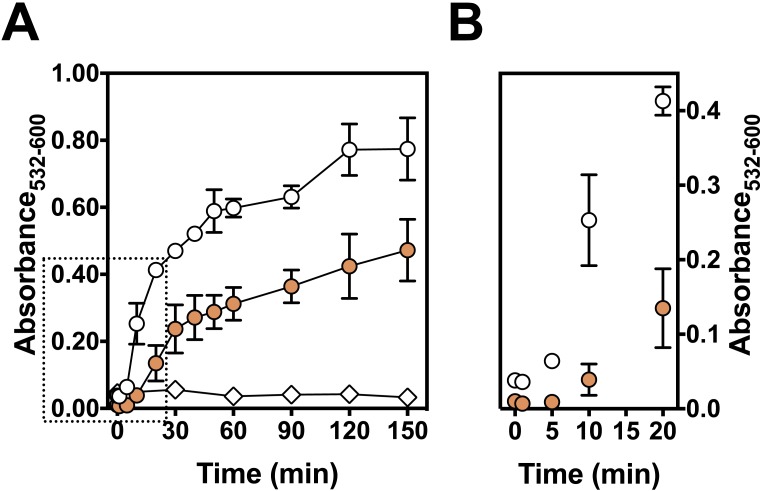
Effect of persimmon extract on lipid peroxidation kinetics. (A) Lipid peroxidation kinetics experiments were performed with 2 mg/ml of persimmon extracts (filled circles; average values from experiments using fruits #6 and #13 individually) or with 0.1 mM BHT (empty diamonds). Experimental conditions were compared with a control condition performed in the absence of extracts (empty circles). Reaction media also contained 10 mM HEPES buffer (pH 7.2), 125 mM KCl, rat liver homogenate (5% v/v), 50 µM Fe(III)-citrate (1:1 ratio) and 50 µM ascorbate. (B) Expansion of the first 20 min.

The kinetics of Fe(III)-citrate mediated lipid peroxidation was determined with 50 µM ascorbate (Fig. 5A). The presence of persimmon extracts extended the lag phase of TBARS formation to about 5–10 min before the onset of the propagation phase (Fig. 5B). Moreover, the addition of persimmon extract reduced both the rate of the propagation phase and maximal levels of TBARS formation (Fig. 5A). The lag phase extension by astringent persimmon extracts have been reported using LDL oxidation method (Katsube et al., 2004). This observation indicates a chain-breaking mechanism, which is consistent with free radical scavenger properties (Halliwell & Gutteridge, 2015). Moreover, the decrease in the propagation rate by persimmon extract indicates that a metal-chelation mechanism could contribute, at least partially, to the antioxidant effect against lipid peroxidation (Halliwell & Gutteridge, 2015).

### Diversity of antioxidant activity among fruits and correlations between 2-DR assay and lipid peroxidation or sugar content

**Figure 6:**
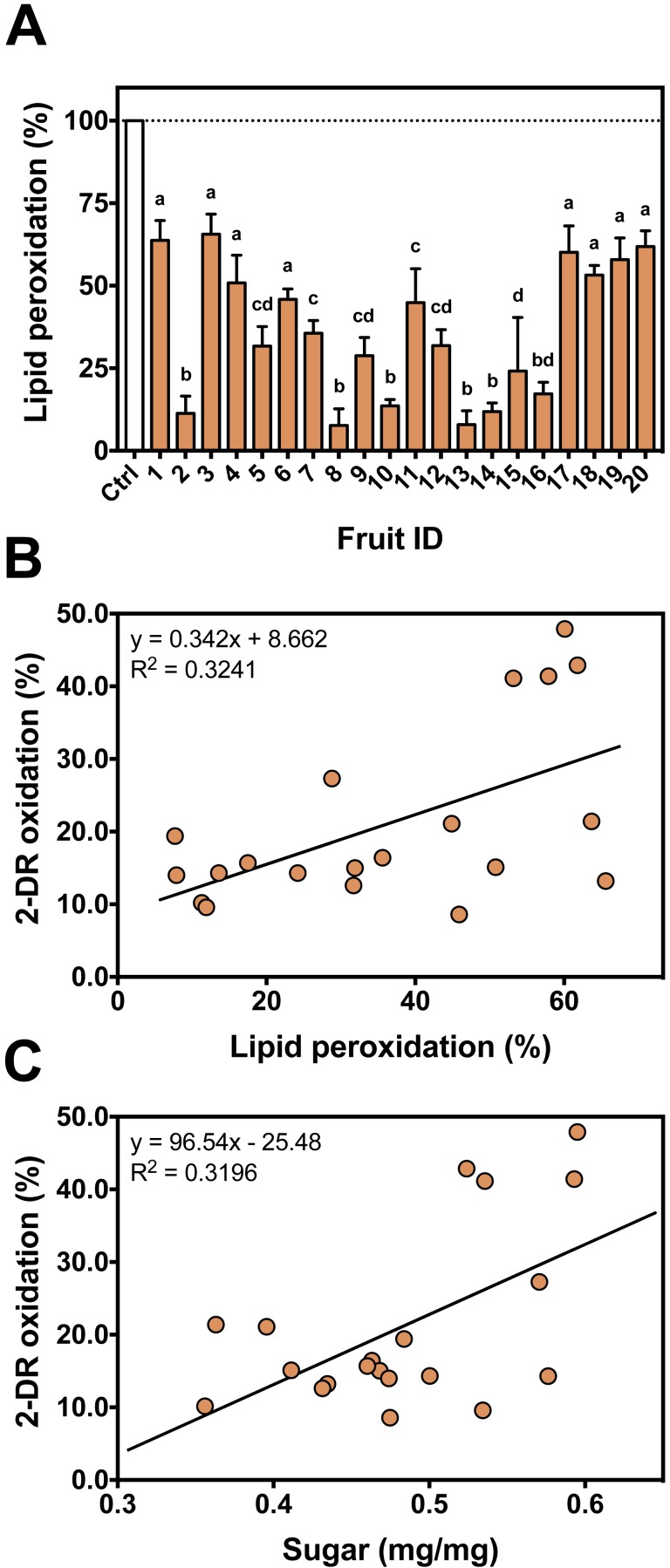
Individual antioxidant activity against iron-induced lipid peroxidation and correlation between assays. (A) Antioxidant profile of the persimmon fruits used in this study against iron-induced lipid peroxidation. Reactions were conducted in 10 mM HEPES buffered media (pH 7.2) containing 125 mM KCl, 5% (v/v) rat liver homogenate, 50 µM Fe(III)-citrate (1:1 ratio), 50 µM ascorbate and 4 mg/mL persimmon extracts. Reactions were started by addition of Fe(III)-citrate to media and carried out for 60 min at room temperature. Values are means + SD (*n* = 6). Distinct letter labels indicate significant difference among values (*p* < 0.05; ANOVA/SNK). (B) Correlation between the protective effects of each persimmon fruit obtained from 2-DR degradation (those depicted in Fig. 2B) and lipid peroxidation assays. (C) Correlation between the total soluble sugar content among persimmon extracts (from all twenty fruits) and their effect on iron-induced 2-DR oxidation (from Fig. 2B). The higher the sugar concentration, the lower the antioxidant action of extracts.

To evaluate antioxidant variability among different fruits against iron-mediated lipid peroxidation, the 20 fruits were individually tested (Fig. 6A). Similar to 2-DR assays, fruits #17 to #20 showed much lower protection against lipid peroxidation. Some fruits presented higher antioxidant effects in hydrophilic media (i.e., 2-DR assay) and poorer effects in hydrophobic conditions (i.e., lipid peroxidation). Such differences can be related to post-harvest treatments process that may produce insoluble tannins (Del Bubba et al., 2009), which still may able to exert antioxidant activity in the lipophilic environments of the lipid peroxidation assay.

Correlation analyses showed that there is a significant (Spearman *R* = 0.4677; *R*^2^ = 0.3241; *p* = 0.0376; 20 XY pairs) and positive correlation among antioxidant profile in the 2-DR assay and that observed in lipid peroxidation experiments (Fig. 6B). A positive correlation suggests that the extracts act as antioxidant in different environments. The observed low *R* value can be explained by different molecular compositions. Fruits containing hydrophilic molecules would be more effective against 2-DR oxidation and those containing hydrophobic molecules would be more effective against lipid peroxidation. Such variability in the composition could be related to the maturation stage of the fruits at harvesting and to the postharvest treatment of the fruits to reduce astringency since maturation and/or postharvest treatments decrease the amount of soluble tannins (Del Bubba et al., 2009). Since fruits were purchased from local markets in three different days without references on harvest date, this variability is expected and should affect the antioxidant profile of the fruits.

Sugar content is considered a marker for the maturation state of fruits (Prasanna, Prabha & Tharanathan, 2007). In persimmons, the increase in the sugar content occurs in parallel with a decrease in tannins concentration, especially the soluble low molecular weight fraction (Del Bubba et al., 2009). We analyzed the total sugar content to better characterize fruit maturation stage of our persimmon fruits purchased in local market. Even though sugar concentration had considerable variation among the 20 fruits, it presented a significant (Pearson *R* = 0.5654; *R*^2^ = 0.3196; *p* = 0.0094; 20 XY pairs) and positive correlation with the extent of 2-DR oxidation (Fig. 6C). This result indicates that the higher the sugar content, the lower the soluble antioxidant activity of persimmon extracts. This is in line with observations by Del Bubba et al. (2009) that soluble radical scavenging activity (DPPH assay) decreases during ripening and after post-harvesting treatments. Interestingly, sugar content did not correlate with anti-peroxidation effect among our 20 fruit extracts (Spearman *R* = 0.0015; *R*^2^ < 0.001; *p* = 0.9950; 20 XY pairs).

Persimmon fruits showed antioxidant activity in the 2-DR degradation assays, in spin trapping EPR study and in the lipid peroxidation. The I_50_ values obtained by aqueous persimmon extracts against 2-DR oxidation (ranging from 0.5 to 1.8 mg/mL) are similar to other fruits such as açaí (Torma et al., 2017), date (Vayalil, 2002) and carissa fruits (Sahreen, Khan & Khan, 2010). In general, the antioxidant capacity of persimmon extracts seems to be lower in a more lipophilic environment. Similar differences of antioxidant capacities between the two assays were observed in *Artemisia nilagirica* fruit, which presented 50% protection at 0.23 mg/mL and 0.47 mg/mL against 2-DR oxidation and lipid peroxidation, respectively (Suseela, Gopalakrishnan & Varghese, 2010).

The individual analyses of each persimmon fruit revealed differences in the antioxidant profile among fruits, indicating that there is a variation in quantity and composition of antioxidant molecules. Studies investigating the antioxidant effects of vegetal extracts used pools of fruits (Vayalil, 2002; Chaudhary et al., 2006; Barreira et al., 2008) and most of them do not mention the number of fruits analyzed. The investigation of separate fruits allowed the assessment and comparison of the variability in antioxidant activity of individual persimmon fruits, for both 2-DR and peroxidation assays. This variability among individual fruits can be attributed to different antioxidant content. As discussed above, individual variation in maturation, which alter levels and composition of antioxidants, could explain the variability.

**Figure 7:**
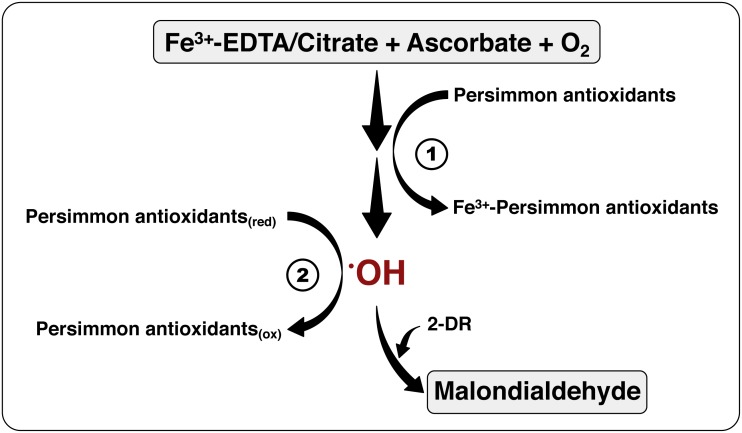
The mechanism of antioxidant action of persimmon extracts. This scheme summarizes the chemical system employed for hydroxyl radical formation, utilizing Fe^3+^-EDTA (or Fe^3+^-citrate), plus ascorbate and oxygen, and detection, by means of 2-DR oxidation, with formation of malonaldehyde (see reactions 1–5 at the ‘Material and Methods’). The antioxidant activity of persimmon extracts may be a result of two mechanisms: (1) metal-chelating activity, which would have inhibited the formation of hydroxyl radicals; and/or (2) radical sequestration, where antioxidants in the extracts react directly with hydroxyl radicals. Our results indicate that antioxidants in pulps of commercial Rama Forte persimmon fruits present, preferentially, mechanism 2.

Tannins and other phenolic substances are abundant in persimmon fruits. These polyphenolic substances may be the main group of substances with antioxidant activity in persimmon fruits (Del Bubba et al., 2009). They can donate hydrogen atoms to free radical molecules, acting as typical radical scavengers. Moreover, they can chelate metal cations, interfering with metal-mediated free radical formation (Andrade et al., 2006; Gu et al., 2008; Perron & Brumaghim, 2009). Soluble tannins in persimmon are largely constituted of *p*-coumaric acid and gallic acid (Gu et al., 2008), which can coordinate with metal ions and prevent ROS production. The observations from the present study (discussed in ‘The 2-DR Degradation Assay’) point towards a scavenger-antioxidant mechanism, rather than metal-chelating antioxidant mechanism (see scheme in Fig. 7), for the commercial persimmon fruits employed herein.

Astringency removal, an usual post-harvest procedure to make persimmon fruits more palatable (Kato, 1990; Vitti, 2009), reduces the content of the low molecular weight polyphenols that contain metal chelating substances, decreasing antioxidant efficiency (Del Bubba et al., 2009). Fruits in this study went through such treatments, which might explain why the absence of metal-chelating antioxidant mechanism in the 2-DR degradation assays. Our results also indicate that the riper fruits, richer in sugar and poorer in tannins, were less effective in suppressing 2-DR oxidative damage. Del Bubba et al. (2009) also observed that more mature fruits had increased levels of glucose and fructose and diminished antioxidant activity.

In our lipid peroxidation assays, we observed a short extension of the lag phase (initiation phase) and a decrease in the log phase (propagation phase) as well as a decrease in the total TBARS formation in the presence of the fruit extracts (see ‘Iron-mediated Lipid Peroxidation’). The high molecular weight tannins are less affected by post-harvest treatments (Del Bubba et al., 2009). These lipophilic tannins, present in the persimmon extracts, may exert a better antioxidant activity in lipophilic environments (i.e., lipid membranes), and contribute to the anti-peroxidation effect observed. One way to solve this issue would be to estimate the total phenolic contents in our samples. In fact, such measurements in vegetable extracts and other biological samples have gained great attention due to the antioxidant activity of phenolic compounds. Often, such measurement is made by means of the colorimetric Folin–Ciocalteu assay. Although widespread, the Folin–Ciocalteu method and its variations are characterized by several limitations. The major limitation is that the assay is rather unspecific, detecting the ability to reduce phosphomolybdic and phosphotungstic acids, and not an actual determination of phenolic (Tan & Lim, 2015). In that regard, there is a range of interfering molecules commonly present in fruit extracts that may strongly affect the outcome of the assay. Those include sugars, ascorbic acid, sulfites, N-amino compounds and N-heterocycles (Ainsworth & Gillespie, 2007; Tan & Lim, 2015). Another confounding factor is the heterogeneity of phenolic compounds in the sample (Singleton, Orthofer & Lamuela-Raventós, 1999). In the case of persimmon fruits, ascorbic acid is present at considerable levels (Kondo, Yoshikawa & Katayama, 2004; Del Bubba et al., 2009) and could be a relevant interfering molecule in the Folin–Ciocalteu assay. In fact, we observed that the EPR signal intensity of ascorbyl radicals formed in reaction mixtures containing 1 mM ascorbate plus 50 µM Fe(III)-citrate (determined as in Dalvi et al., 2017) and persimmon fruit extracts was increased as the amount of extracts was increased (Table S1). This happened also with no iron addition to reaction mixtures, indicating a high concentration of ascorbic acid in the persimmon fruits. Therefore, we opt not to include Folin–Ciocalteu assays in our study.

## Conclusions

Finally, the different approaches reported here indicate that the antioxidant activity of aqueous persimmon extract derives predominantly from its ability to scavenge free radicals (see Fig. 7). This was unexpected in regard to an astringent fruit strain rich in tannins, whose chelating activity is well known. The fruits used here, however, were obtained from commercial suppliers that expose fruits to post-harvest treatment prior to selling (Vitti, 2009). Such treatment greatly reduces the tannin content (Salvador et al., 2007; Del Bubba et al., 2009), improving palatability but decreasing metal-chelating activity. Still, these are the Rama Forte persimmon fruits available for Brazilian consumers and they present a significant antioxidant activity. Finally, we believe that researchers interested in food redox chemistry may benefit from the experimental approach employed here to distinguish metal-chelating from radical-scavenging antioxidant activity when studying other fruits.

##  Supplemental Information

10.7717/peerj.5223/supp-1 Table S1 Ascorbyl radical intensity in different persimmon extract concentrations measured by EPR as in Dalvi et al. (2017)

10.7717/peerj.5223/supp-2 Supplemental Information 1 Raw data tables

## Additional Information and Declarations

### Data Availability

The following information was supplied regarding data availability: The raw data are provided in Supplemental File.
